# IgE Activates Monocytes from Cancer Patients to Acquire a Pro-Inflammatory Phenotype

**DOI:** 10.3390/cancers12113376

**Published:** 2020-11-15

**Authors:** Mano Nakamura, Elmira Amiri Souri, Gabriel Osborn, Roman Laddach, Jitesh Chauhan, Chara Stavraka, Sara Lombardi, Anna Black, Atousa Khiabany, Duaa O. Khair, Mariangela Figini, Anna Winship, Sharmistha Ghosh, Ana Montes, James F. Spicer, Heather J. Bax, Debra H. Josephs, Katie E. Lacy, Sophia Tsoka, Sophia N. Karagiannis

**Affiliations:** 1 St. John’s Institute of Dermatology, School of Basic & Medical Biosciences, King’s College London, Tower Wing, 9th Floor, Guy’s Hospital, London SE1 9RT, UK; mano.nakamura@kcl.ac.uk (M.N.); gabriel.osborn@kcl.ac.uk (G.O.); roman.laddach@kcl.ac.uk (R.L.); jitesh.chauhan@kcl.ac.uk (J.C.); chara.stavraka@kcl.ac.uk (C.S.); sara.lombardi@gstt.nhs.uk (S.L.); anna.black@gstt.nhs.uk (A.B.); AXK1104@student.bham.ac.uk (A.K.); duaa.khair@ouh.nhs.uk (D.O.K.); heather.bax@kcl.ac.uk (H.J.B.); debra.josephs@gstt.nhs.uk (D.H.J.); katie.lacy@gstt.nhs.uk (K.E.L.); 2 Department of Informatics, Faculty of Natural & Mathematical Sciences, King’s College London, London WC2B 4BG, UK; elmira.amiri@kcl.ac.uk (E.A.S.); sophia.tsoka@kcl.ac.uk (S.T.); 3 School of Cancer & Pharmaceutical Sciences, King’s College London, Guy’s Hospital, London SE1 9RT, UK; james.spicer@kcl.ac.uk; 4 Biomarker Unit, Department of Applied Research and Technology Development, Fondazione, IRCCS Istituto Nazionale dei Tumouri Milano, 20133 Milan, Italy; mariangela.figini@istitutotumori.mi.it; 5 Department of Medical Oncology and Clinical Oncology, Guy’s and St Thomas’ NHS Foundation Trust, London SE1 9RT, UK; anna.winship@gstt.nhs.uk (A.W.); sharmistha.ghosh@gstt.nhs.uk (S.G.); ana.montes@gstt.nhs.uk (A.M.); 6 Breast Cancer Now Research Unit, School of Cancer & Pharmaceutical Sciences, King’s College London, Guy’s Cancer Centre, London SE1 9RT, UK

**Keywords:** IgE, monocytes, FcεRI, cancer, cancer immunotherapy, AllergoOncology, cytotoxicity, cross-linking

## Abstract

**Simple Summary:**

When activated by tumour antigen-specific IgEs, monocytes may contribute to the restriction of cancer growth in animal models of cancer. In this study, we investigated the effects of IgE stimulation on the activation state of human monocytes from healthy subjects and from patients with cancer. Cross-linking of cognate Fc receptors by IgE on human monocytes potentiated: (a) upregulation of activatory and down regulation of regulatory monocyte cell surface markers; (b) phosphorylation of intracellular protein kinases in monocytes previously described to be downstream of the mast cell and basophil FcεRI signalling pathway; (c) ovarian cancer patient monocyte-mediated cytotoxic killing of tumour cells and release of pro-inflammatory mediators, potentially associated with favourable patient survival. IgE can therefore activate human monocytes to acquire a pro-inflammatory phenotype capable of mediating effector functions against tumour cells. This may contribute to the mechanism of cancer immunotherapy using IgE antibodies.

**Abstract:**

IgE contributes to host-protective functions in parasitic and bacterial infections, often by monocyte and macrophage recruitment. We previously reported that monocytes contribute to tumour antigen-specific IgE-mediated tumour growth restriction in rodent models. Here, we investigate the impact of IgE stimulation on monocyte response, cellular signalling, secretory and tumour killing functions. IgE cross-linking on human monocytes with polyclonal antibodies to mimic formation of immune complexes induced upregulation of co-stimulatory (CD40, CD80, CD86), and reduced expression of regulatory (CD163, CD206, MerTK) monocyte markers. Cross-linking and tumour antigen-specific IgE antibody-dependent cellular cytotoxicity (ADCC) of cancer cells by cancer patient-derived monocytes triggered release of pro-inflammatory mediators (TNFα, MCP-1, IL-10, CXCL-10, IL-1β, IL-6, IL-23). High intratumoural gene expression of these mediators was associated with favourable five-year overall survival in ovarian cancer. IgE cross-linking of trimeric FcεRI on monocytes stimulated the phosphorylation of intracellular protein kinases widely reported to be downstream of mast cell and basophil tetrameric FcεRI signalling. These included recently-identified FcεRI pathway kinases Fgr, STAT5, Yes and Lck, which we now associate with monocytes. Overall, anti-tumour IgE can potentiate pro-inflammatory signals, and prime tumour cell killing by human monocytes. These findings will inform the development of IgE monoclonal antibody therapies for cancer.

## 1. Introduction

Monocytes engender multiple roles in homeostatic and disease processes, including in tissue repair [1], allergic inflammation [2], and bacterial and parasite infection clearance [3,4,5]. They also reportedly exert pro- and anti-tumour functions [6]. Monocytes are also known to express IgE Fc receptors (FcεRs) [7,8,9]. IgE class antibodies participate in allergic disease pathology, and exert host protective functions in parasitic and bacterial infections, often by recruitment and stimulation of monocytes and macrophages [3,4,5]. However, the response of human monocytes to IgE stimulation remains unclear.

While IgG is a well-established antibody modality for the treatment of cancer, IgE has not been used for cancer therapy until now, with the first clinical trial of an IgE class therapeutic candidate for oncology nearing completion [10]. Several attributes of IgE may provide an alternative approach to activating human immune effector cells against cancer in comparison to IgG. These include IgE’s high affinity for cognate Fc receptors (FcεRs) (2 to 5-fold greater than IgG affinity for FcγRs) [11] and strong retention by FcεR-expressing immune effector cells, including mast cells, basophils, eosinophils, dendritic cells, monocytes and macrophages [9,11,12,13,14,15,16,17,18,19,20].

Several studies have demonstrated that when designed to recognize tumour-associated antigens, IgE antibodies can activate immune cells, such as monocytes [8,15] and macrophages [21,22], to engender anti-tumour immunity by effector functions. Particularly, monocytes may play important roles in IgE-mediated anti-tumour efficacy. MOv18 IgE, specific for the ovarian cancer-associated antigen folate receptor alpha (FRα) enhanced the survival of patient-derived orthotopically-grown ovarian cancer xenograft-bearing mice reconstituted with human immune cells, but only when monocytes were present in the immune effector cell population [8]. In an immunocompetent syngeneic rat tumour model, systemic treatment with MOv18 IgE was associated with pronounced infiltration of rat CD68+ monocytes and macrophages into lung-resident tumours. Furthermore, monocytes and macrophages in IgE-treated rat tumours showed enhanced intracellular TNFα and IL-10 expression, and significantly upregulated TNFα, MCP-1 and IL-10 levels in the bronchoalveolar lavage (BAL) fluid, compared with IgG- or PBS control-treated groups [12,23]. This cytokine profile was also significantly elevated in supernatants of antibody-dependent cellular cytotoxicity (ADCC) of human IGROV1 ovarian tumour cells by human primary monocytes treated with MOv18 IgE, compared with non-specific IgE [12,23,24]. These results suggest that IgE-monocyte-mediated anti-tumour effects may be delivered via IgE Fc-mediated ADCC and an immune mediator milieu, including the pro-inflammatory TNFα and the monocyte chemoattractant MCP-1, contributing to an activation cascade analogous to that triggered in IgE-mediated protective responses against parasite infection [25].

Here, we hypothesised that IgE may engender fundamental changes in monocytes’ activation state, their signalling and secreted immune mediator network. To evaluate the immunological environment associated with IgE-dependent monocyte-mediated functions, we investigated the cytokine and chemokine milieu, signalling pathways and anti-tumour functions triggered by IgE stimulation of human monocytes derived from healthy subjects and from patients with ovarian cancer.

## 2. Results

### 2.1. Cross-Linking of IgE on Human Monocytes Induces Pro-Inflammatory Cell Surface Receptor Expression Profiles

**Figure 1:**
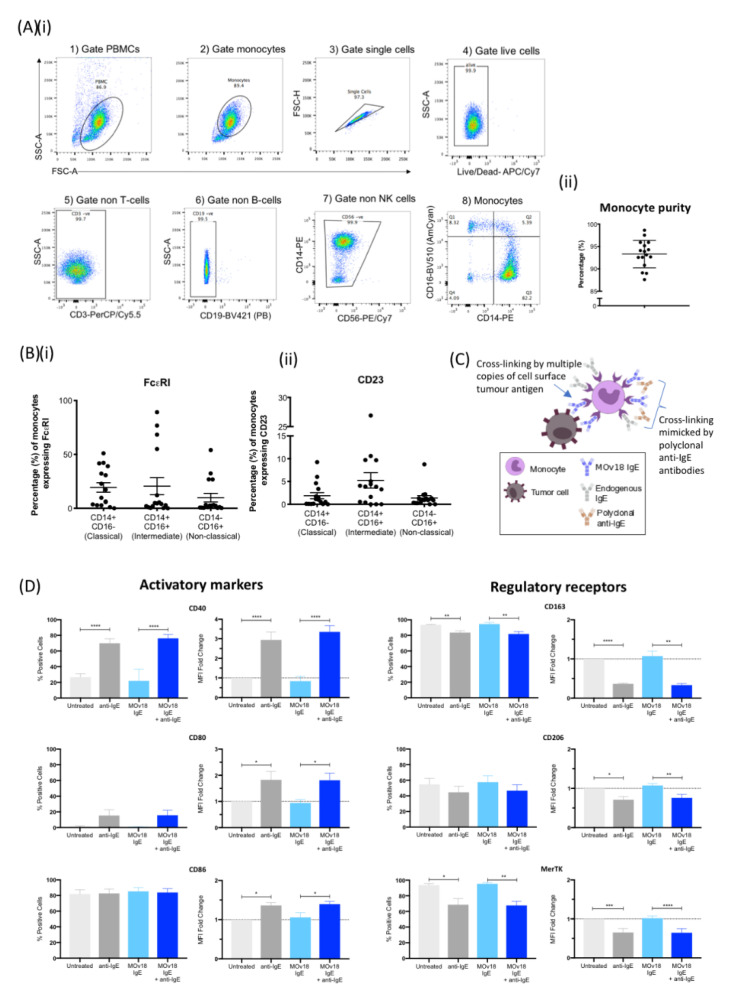
IgE cross-linking on the surface of monocytes triggers upregulation of pro-inflammatory cell surface marker expression and downregulation of regulatory cell surface marker expression. (**A**) (**i**) Sequential gating strategy of monocyte population isolated from fresh healthy volunteer blood: gate (1) PBMC and (2) monocytes, then gate (3) single cells, (4) live cells, (5) non-T cells (CD3), (6) non-B cells (CD19), (7) non-NK cells (CD56) and (8) monocytes (X axis: CD14, Y axis: CD16) and (**ii**) % purity of isolated monocytes ± SD (*n* = 16). (**B**) Expression of IgE Fc receptors (**i**) FcεRI and (**ii**) CD23 per monocyte subset (classical, intermediate, non-classical). (**C**) Schematic representation of IgE antibody cross-linking when monocytes engage with target antigen-expressing tumour cells and cross-linking mimicked by addition of polyclonal anti-IgE antibodies. (**D**) Percentage of positive cells (left), and fold-change in mean fluorescence intensity (MFI) (right) of activatory (left panel) and regulatory receptor (right panel) surface markers upon IgE cross-linking on the surface of healthy volunteer monocytes. Fold-change was calculated in relation to “untreated” (light grey). Error bars represent standard error of mean (SEM) unless stated otherwise (CD40, CD80, CD86, and CD206, *n* = 9; CD163 and MerTK, *n* = 3) (independent experiments). A one-way ANOVA with Tukey’s post-hoc test was performed to assess significance (* *p* < 0.05; ** *p* < 0.01; *** *p* < 0.001; **** *p* < 0.0001).

Human peripheral blood monocytes (CD14+CD56-CD3-CD19-) were identified (Figure 1(Ai)) with 93% mean purity (Figure 1(Aii)). Expression of the high-affinity (FcεRI) and the low-affinity (CD23) IgE receptors amongst monocyte subsets were assessed (Figure 1(Bi,ii)), and overall FcεR expression was confirmed in accordance with previous studies (Figure S1A,B). Due to the high affinity and slow dissociation rate between IgE and FcεRI, we expected FcεRI to be partially occupied with endogenous IgE. Consistent with previous findings [8,15,22], monocytes could bind exogenous MOv18 IgE on remaining unoccupied FcεRs (Figure S1C).

We evaluated whether IgE cross-linking on human monocytes could influence cell surface expression of markers of classical and alternative immune activation (Figure 1C). Monocytes derived from healthy volunteers were either untreated or incubated with anti-tumour MOv18 IgE. Additionally, IgE-FcεR cross-linking was triggered by polyclonal anti-human IgE to mimic immune complex formation. Cross-linking FcεRs, either partially occupied by endogenous IgEs or saturated by addition of MOv18 IgE, led to significant upregulation of co-stimulatory and activatory monocyte markers CD40, CD80 and CD86 expression (Figure 1D, left panel). Simultaneously, IgE cross-linking triggered significant decreases in monocyte regulatory markers CD163, CD206 and MerTK (Figure 1D, right panel).

These findings suggest that IgE cross-linking may skew monocytes towards a pro-inflammatory and activated state.

### 2.2. Cross-Linking of FcεR-Bound IgE Increases Production of Pro-Inflammatory Mediators by Monocytes

**Figure 2:**
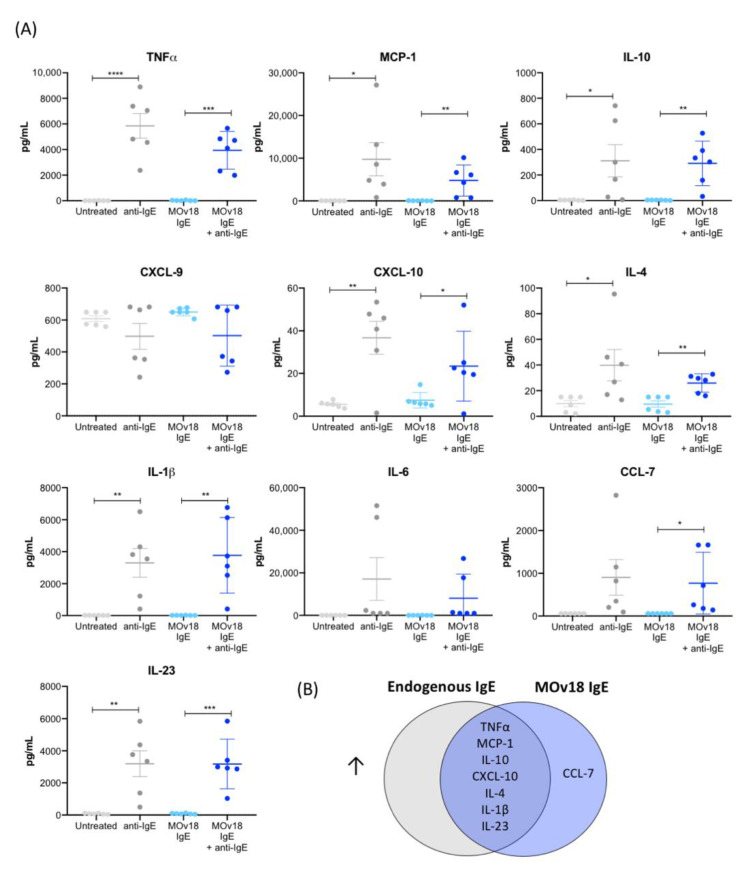
IgE cross-linking on the surface of monocytes triggers increased pro-inflammatory cytokine and chemokine release. (**A**) Cytokine and chemokine production measured in cell culture supernatants following cross-linking of IgE on the surface of primary monocytes isolated from healthy volunteer blood (*n* = 6). (**B**) Venn diagram of the cytokines and chemokines that were significantly upregulated upon cross-linking of endogenous or MOv18 IgE, or both. Error bars represent standard error of mean (SEM) (independent experiments). A one-way ANOVA with Tukey’s post-hoc test was performed to assess significance (* *p* < 0.05; ** *p* < 0.01; *** *p* < 0.001; **** *p* < 0.0001).

IgE treatment has been associated with pro-inflammatory mediator release in the tumour microenvironment in rodent models [12]. Thus, we investigated whether human monocytes could be stimulated by IgE to release such mediators. In concordance with previous findings, IgE cross-linking triggered secretion of cytokines known to be involved in immune responses to parasite infection and Th1 immunity. TNFα and MCP-1 were upregulated by cross-linking of FcεRs when occupied with either endogenous IgE only or when saturated through addition of MOv18 IgE (Figure 2A). IgE stimulation triggered release of the pro-inflammatory Th1-related chemokine CXCL-10 by both cross-linking conditions. Furthermore, Th2-related (IL-10, IL-4), classically-activated (M1) macrophage (IL-1β) and Th17-related mediators (IL-23), were significantly increased with both endogenous IgE cross-linking and MOv18 IgE cross-linking. CCL-7, another mediator known to participate in anti-parasitic and infection responses, was upregulated upon MOv18 IgE cross-linking only, while the classical inflammatory mediator IL-6 was not significantly changed (Figure 2 and Figure S2). There were no differences in the concentrations of cytokines between endogenous IgE cross-linking and MOv18 IgE cross-linking conditions.

Together, these findings suggest that IgE stimulation on human monocytes triggered release of pro-inflammatory mediators (TNFα, MCP-1, IL-10, CXCL-10, IL-4, IL-1β and IL-23) (Figure 2B).

### 2.3. Cross-Linking IgE on the Monocyte Surface Activates Protein Kinases Downstream of the FcεRI and Activation of Several Immune Pathways

**Figure 3:**
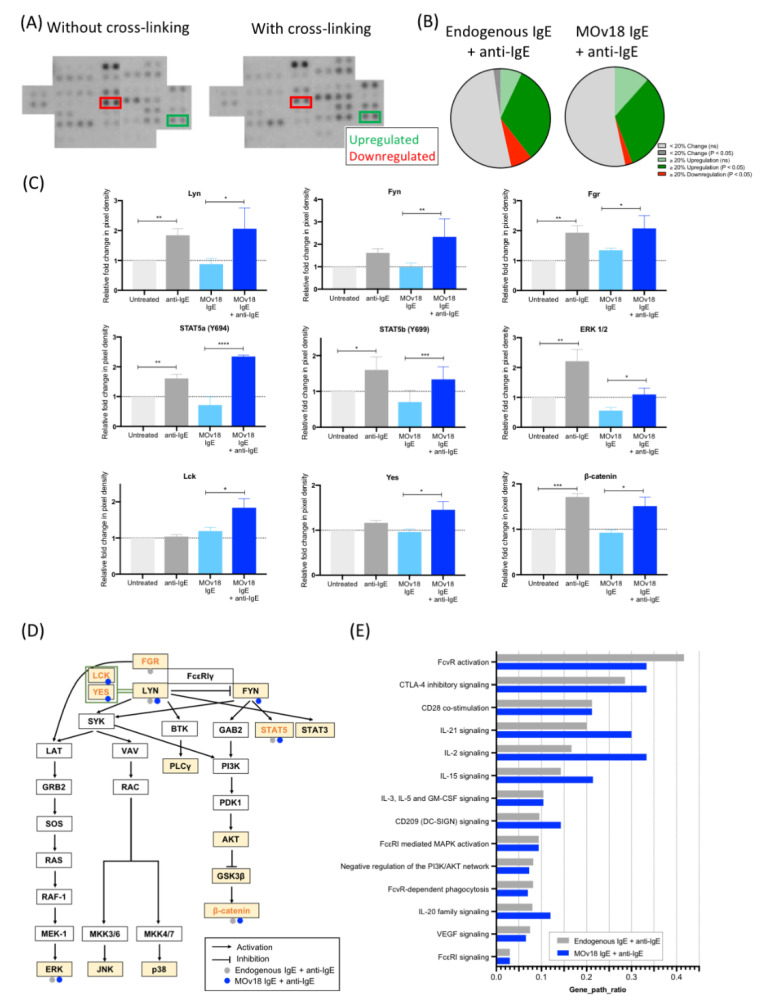
IgE cross-linking on the surface of monocytes activates protein kinases downstream of FcεRIα and other immune activatory pathways. (**A**) Images from Proteome Profiler Human Phospho-Kinase Array with and without IgE cross-linking on the surface of healthy volunteer-derived primary monocytes. Each kinase is spotted in duplicate. (**B**) Summary of changes in protein kinase phosphorylation after cross-linking endogenous IgE (left) and MOv18 IgE (right). (**C**) Pixel density was analysed and expressed as fold-change relative to “untreated” (light grey) (Y = 1) comparing samples subjected to treatment (+ anti-IgE (dark grey); MOv18 IgE (light blue); MOv18 IgE + anti-IgE (dark blue)). (**D**) Schematic of FcεRI pathway network in monocytes generated via the Kyoto Encyclopedia of Genes and Genomes (KEGG) database. Kinases with upregulated phosphorylation upon cross-linking of endogenous IgE (grey) or MOv18 IgE (blue) are indicated. Highlighted in yellow are kinases analysed with the Human Phospho-Kinase Antibody Array. Orange-lettered kinases are newly added onto the pathway based on our observations. (**E**) Other pathways that may be influenced upon IgE cross-linking on the monocyte surface were explored using Reactome pathway enrichment, by studying genes of kinases showing ≥20% change in phosphorylation following IgE stimulation. Error bars represent standard error of mean (SEM) (*n* = 3 independent experiments). A one-way ANOVA with Tukey’s post-hoc test was performed to assess significance (* *p* < 0.05; ** *p* < 0.01; *** *p* < 0.001; **** *p* < 0.0001).

IgE engagement and FcεRI downstream signalling have been widely studied in mast cells [26,27], but not in monocytes. Here, kinase phosphorylation levels were investigated in monocytes after cross-linking of FcεRs partially occupied by endogenous IgE or saturated by MOv18 IgE. Kinase phosphorylation was measured in a phospho-kinase array (Figure 3A), and values were represented as fold-changes in pixel density in relation to the unstimulated “no antibody” control. Of the 43 protein kinases studied, ~40% showed enhanced phosphorylation of ≥20% change with one or both IgE cross-linking conditions (green segments, Figure 3B). We found protein kinases described to be downstream of the tetrameric FcεRI on mast cells and basophils to be phosphorylated with IgE stimulation on monocytes: Lyn and Fgr showed a two-fold increase in phosphorylation with endogenous IgE and with additional MOv18 IgE cross-linking (Figure 3C). Fyn phosphorylation, also a known FcεRI-related kinase, was significantly upregulated by two-fold with MOv18 IgE cross-linking as was phosphorylation of Lck and Yes, which can substitute Fyn [28,29,30] (Figure 3C). The phosphorylation of other kinases not classically known to belong to the tetrameric FcεRI pathway, such as STAT5a, STAT5b, and β-catenin, was also increased by 1.3 to 2.3-fold upon IgE cross-linking. From these findings, the monocytic-FcεRI signalling pathway was mapped and updated based on the equivalent network in mast cells and basophils (Figure 3D, Table S1). Furthermore, Reactome pathway enrichment revealed other potential signalling pathways, such as FcγR activation and CD28 co-stimulation, largely belonging to immune cell activation, stimulation, and antigen presentation processes, to be implicated following IgE-FcεRI cross-linking (Figure 3E, Table S2).

Overall, IgE cross-linking on monocytes triggered increased phosphorylation of protein kinases, already shown to be downstream of FcεRI in mast cells and basophils, but also of recently-identified kinases such as Fgr, STAT5, Yes, and Lck, which we now associate with FcεRI pathway signalling in monocytes.

### 2.4. IgE Potentiates Cytotoxic Killing of Tumour Cells and Immune Activatory Functions of Monocytes from Healthy Volunteers and Ovarian Cancer Patients

**Figure 4:**
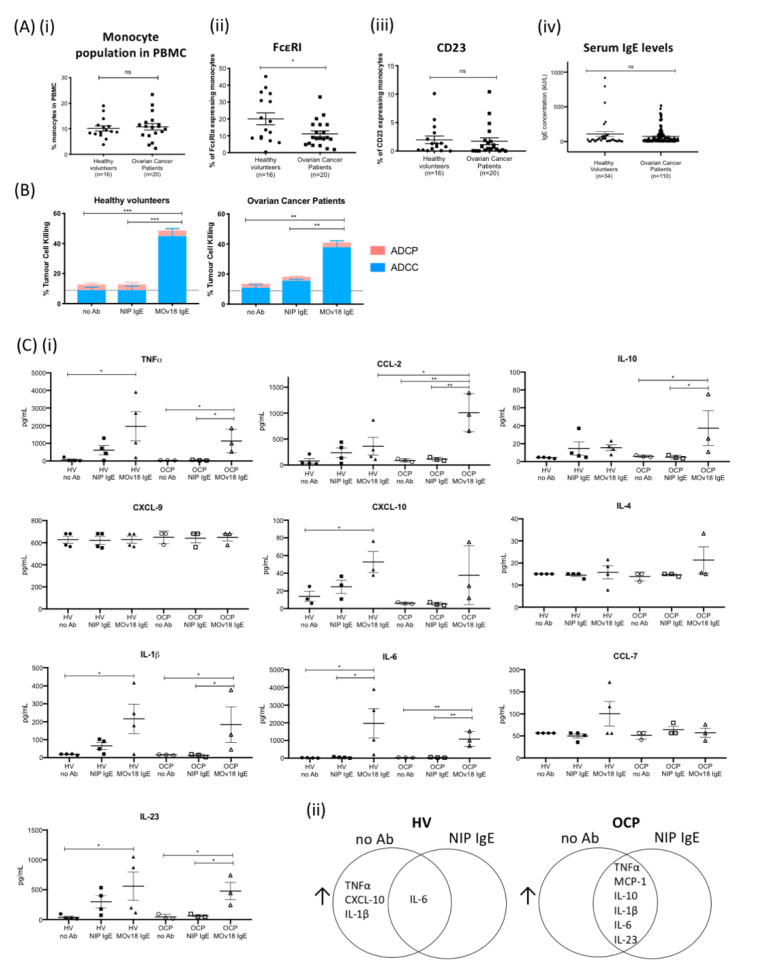
IgE potentiates cancer cell antibody-dependent cellular cytotoxicity (ADCC) and immune mediator release by monocytes from healthy volunteers and ovarian cancer patients. (**A**) (**i**) Proportions of monocytes within total PBMC of healthy volunteers and ovarian cancer patients. Comparison of (**ii**) FcεRI- and (**iii**) CD23-expressing monocyte proportions in healthy volunteers (*n* = 16) and patients (*n* = 20). (**iv**) Total serum IgE concentration measured in healthy volunteers (*n* = 34) and patients with ovarian cancer (*n* = 110). (**B**) MOv18 IgE potentiated in vitro killing of target IGROV1 ovarian cancer cells (compared with no antibody (no Ab) and isotype (NIP IgE) controls) by primary monocytes isolated from healthy volunteers (left, *n* = 4) and patients (right, *n* = 3) (independent experiments). (**C**) Cytokine and chemokine levels measured in cell culture supernatants from IgE-mediated ADCC/ADCP assays with primary monocytes (Luminex). (**i**) Quantitative analyses of immune mediators measured in the supernatants of ADCC/ADCP assays with monocytes from healthy volunteers (HV) (*n* = 4) and from patients with ovarian cancer (OCP) (*n* = 3) as effector cells (data from independent experiments). (**ii**) Venn diagrams of secreted mediators increased in supernatants from ADCC/ADCP assays with primary monocytes from healthy volunteers (left, *n* = 4) and from patients (right, *n* = 3) as effector cells: comparisons between MOv18 IgE treatment versus no antibody (no Ab) and MOv18 IgE treatment versus isotype NIP IgE controls (NIP IgE); upregulated cytokines by MOv18 IgE versus both NIP IgE and no Ab controls are shown for each human donor group. Error bars represent standard error of mean (SEM) of independent experiments. (**A**) A Student’s *t*-test and (**B**,**C**) one-way ANOVA with Tukey’s post-test were performed to assess significance (* *p* < 0.05; ** *p* <0.01; *** *p* < 0.001).

How the IgE/FcεR axis affects functional attributes of human monocytes in healthy subjects and cancer patients is unknown. In ovarian cancer patients and female healthy subjects, the proportions of total monocytes in peripheral blood mononuclear cells were similar (~10%) (Figure 4A). A smaller proportion of monocytes expressed FcεRI in patients, while CD23 expression levels were similar in both groups (Figure 4A). While it has been reported that FcεRI levels on mast cells adjust to the levels of IgE exposure, FcεRI levels did not increase upon IgE stimulation of human monocytes ex vivo, while stimulation with IL-4 independently of IgE was sufficient to upregulate CD23 cell surface expression (Figure S3), in agreement with previous reports [31,32,33]. Furthermore, serum IgE levels were similar in healthy volunteers (*n* = 34) and patients (*n* = 110) (Figure 4A, Table S3).

We next evaluated whether circulating monocytes from patients and healthy subjects could mediate ex vivo tumour cell killing by IgE, namely MOv18 IgE or non-specific NIP IgE isotype control. Primary monocytes from healthy volunteers and ovarian cancer patients mediated ADCC of IGROV1 cells (37% and 22% above NIP IgE controls, respectively) (Figure 4B). In supernatants from tumour cell killing assays, we found a similar immune mediator profile (TNFα, MCP-1, IL-10, CXCL-10, IL-1β, IL-6, and IL-23) to that previously detected with IgE cross-linking on the surface of monocytes. This immune signature was associated with both patient-derived and healthy volunteer monocyte-mediated ADCC (Figure 4C and Figure S4). Similarly, human U937 monocytic cells, which lack endogenous IgE bound on IgE Fc receptors prior to stimulation with anti-tumour IgE, also upregulated TNFα, MCP-1, and IL-10 expression with MOv18 IgE-dependent cytotoxic killing (ADCC) of tumour cells (Figure S5).

Therefore, monocytes from both healthy subjects and cancer patients can be activated by tumour antigen-specific IgE to kill tumour cells and to secrete pro-inflammatory mediators.

### 2.5. IgE-Stimulated Monocytes Can Secrete TNFα, MCP-1 and IL-10

Upregulation of TNFα, MCP-1 and IL-10 has been consistently observed in this study and in previous in vitro and in vivo models of cancer following IgE immunotherapy [12]. We therefore aimed to delineate the conditions required for the release of each mediator triggered by IgE cross-linking and IgE Fc-mediated tumour cell killing.

**Figure 5:**
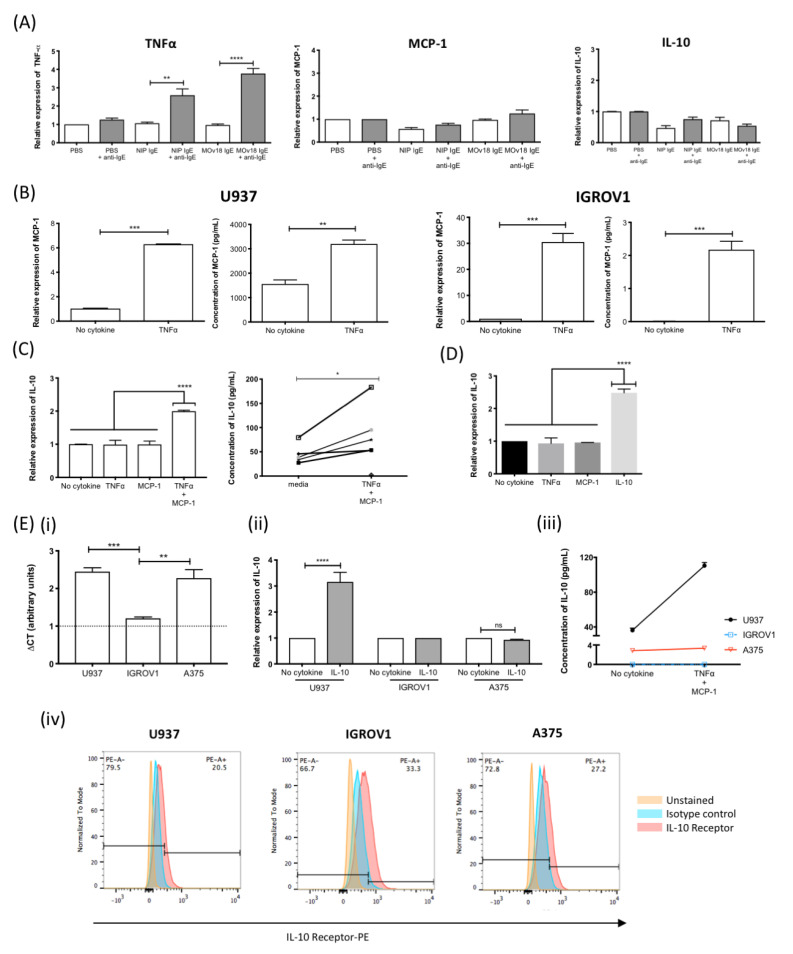
IgE cross-linking on monocytic cells induces a TNFα/MCP-1/IL-10 cascade. (**A**) TNFα, MCP-1 and IL-10 mRNA expression by human monocytic U937 cells treated with buffer alone (PBS) or IgE antibodies (NIP IgE or MOv18 IgE) (white), or IgE cross-linked with polyclonal antibody (grey) (*n* = 4). (**B**) MCP-1 mRNA and protein expression by U937 monocytes (left) and human ovarian IGROV1 (right) cells following TNFα stimulation (*n* = 4). (**C**) IL-10 mRNA expression (left) and secretion (right) by U937 cells following TNFα+MCP-1 combined stimulation (*n* = 5). (**D**) IL-10 mRNA expression by U937 cells following IL-10 stimulation (*n* = 5). (**E**) Comparison of (**i**) baseline IL-10 expression (CT value units; *n* = 2), (**ii**) IL-10 mRNA expression following stimulation with and without IL-10 (*n* = 2), (**iii**) IL-10 secretion following TNFα + MCP-1 combined stimulation (*n* = 2), and (**iv**) IL-10 receptor expression of U937, IGROV1, and human melanoma A375 cell by flow cytometric analysis. Fold-change was calculated in relation to unstimulated cells. Error bars represent standard error of mean (SEM) of independent experiments. One-way ANOVA with Tukey’s post-test and Student’s *t*-test was performed to assess significance (* *p* < 0.05; ** *p* < 0.01; *** *p* < 0.001; **** *p* < 0.0001).

Cross-linking IgE antibodies of various antigen specificities on the surface of human monocytic U937 cells resulted in up to three-fold increase in TNFα expression. However, IgE cross-linking alone did not trigger MCP-1 or IL-10 upregulation (Figure 5A). When monocytic cells were stimulated with TNFα, this resulted in a six-fold upregulation of mRNA expression and a two-fold increased secretion of MCP-1 (Figure 5B, left). Similarly, TNFα upregulated MCP-1 on human ovarian cancer IGROV1 cells (Figure 5B, right). Furthermore, when monocytes were stimulated with TNFα and MCP-1 combined, IL-10 expression and secretion increased by two-fold and 2.5-fold, respectively (Figure 5C). Stimulating monocytic cells with IL-10 alone also triggered a 2.5-fold increase in IL-10 expression (Figure 5D, Figure S6). To determine the cellular source of IL-10, baseline IL-10 mRNA expression in monocytic U937, ovarian cancer IGROV1 and an additional cancer cell line (melanoma A375) was interrogated by qPCR. IGROV1 cells had significantly lower baseline IL-10 expression compared with U937 or A375 (Figure 5(Ei)). Only U937 cells responded to IL-10 (Figure 5(Eii)) or TNFα and MCP-1 combination stimulation (Figure 5(Eiii)) to upregulate IL-10, despite all cell types expressing the IL-10 receptor (Figure 5(Eiv)).

Together, these data suggest that a TNFα-MCP-1-IL-10 axis is potentiated by IgE engagement on monocytes.

### 2.6. IgE-Mediated Immune Mediator Signatures Are Associated with Favorable Overall Survival in Ovarian Cancer

**Figure 6:**
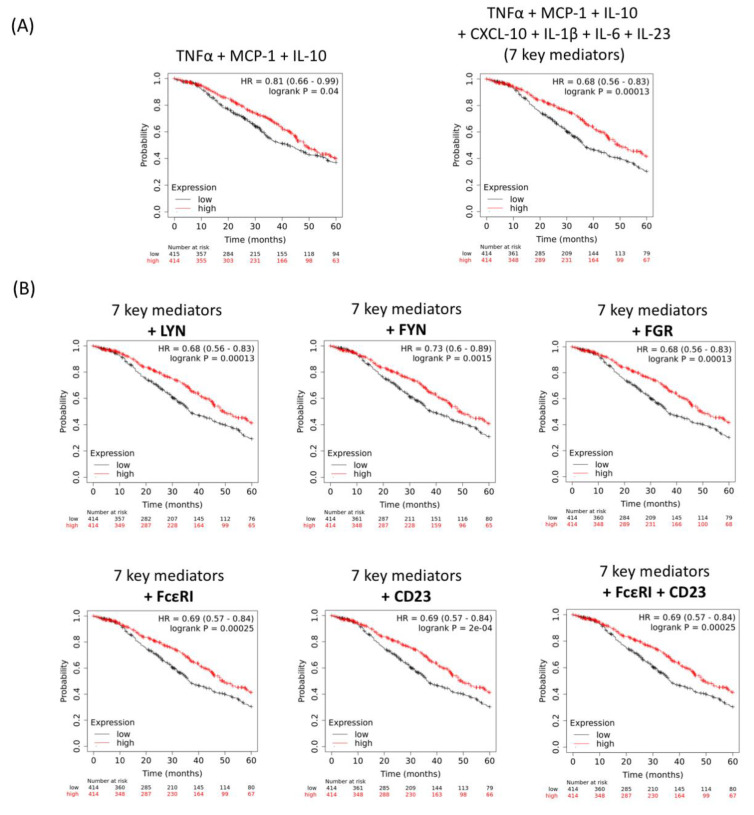
IgE-mediated cytokines, chemokines and protein kinase signatures may be associated with favourable patient survival in ovarian cancer. Kaplan–Meier survival curves showing high intratumoural gene expression (red) of immune mediator combinations, linked with IgE stimulation in our study, which is associated with improved five-year overall survival in 1656 ovarian cancer patients. (**A**) TNFα + MCP-1 + IL-10 and TNFα + MCP-1 + IL-10 + CXCL-10 + IL-1β + IL-6 + IL-23 (7 key mediators). (**B**) As shown above, 7 key mediators + LYN; 7 key mediators + FYN; 7 key mediators + FGR; 7 key mediators + FcεRI; 7 key mediators + CD23; (7 key mediators) + FcεRI + CD23.

We next investigated whether intratumoural expression of cytokine signatures and protein kinases associated with IgE stimulation of monocytes may be linked to ovarian cancer patient outcomes. Kaplan–Meier analyses in a cohort of 1656 patients were conducted to evaluate five-year overall survival. High intratumoural gene expression of a combination of TNFα, MCP-1, and IL-10, and TNFα, MCP-1, IL-10, CXCL-10, IL-1β, IL-6 and IL-23 (seven key mediators) was associated with better patient survival in comparison to patients with low intratumoural gene expression of these mediators (HR = 0.81, *p* = 0.04; HR = 0.68, *p* = 0.00013, respectively) (Figure 6A). Consistently, high intratumoural gene expression of the protein kinases LYN, FYN, FGR and FcεRs (FcεRI and CD23) each combined with the seven key mediators was also associated with favourable overall survival (Figure 6B). This suggested that IgE-mediated FcεR signalling and immune signatures were associated with improved overall survival in ovarian cancer.

## 3. Discussion

IgE antibodies directed against cancer-associated antigens have been shown to restrict tumour growth in several in vivo cancer models [8,12,15,22,23,34]. This concept has been translated to the clinic, and an ongoing first-in-human phase I clinical trial of MOv18 IgE targeting folate receptor-alpha (FRα) (NCT02546921, www.clinicaltrials.gov) recently reported promising interim data in patients with ovarian cancer [10]. IgE is an emerging therapeutic platform to complement IgG-based therapies in oncology. FcεRs with high affinity for IgE are expressed on different immune cells including monocytes. Monocytes can orchestrate IgE-mediated anti-tumour immunity [8,14,35]. However, the ability of IgE to influence the signalling, secretory, and effector capacities of monocytes in healthy subjects and in patients with cancer remains unclear. Here, we provide evidence that IgE exerts a significant switch in human monocytes to acquire pro-inflammatory activation features.

We studied the effects of IgE stimulation on the surface of primary human monocytes by cross-linking FcεRs either partially engaged with endogenous IgE or saturated by addition of exogenous MOv18 IgE to mimic antibody-immune complex formation. IgE cross-linking induced a phenotypic shift in monocyte surface marker expression suggestive of pro-inflammatory immune activation. This included a significant upregulation of co-stimulatory molecules, CD40, CD80 and CD86 (Figure 1). These markers signify reciprocal monocyte-mediated activation of anti-tumour immunity such as via T cell co-stimulation and priming [36,37,38]. Consistent with this, IgE/FcεRI-dependent antigen uptake has been found to induce antigen-specific T cell effector function and to increase tumour-free survival in melanoma-bearing mice [17]. Interestingly, when IgE was cross-linked on dendritic cells, CD86 expression was not upregulated [39,40]. Reciprocally, CD40 engagement by Th1 cells heightens the tissue destructive capacity of monocytes through upregulating pro-inflammatory mediators such as TNFα, and IL-1β, also detected with IgE stimulation of monocytes in our study [36]. Simultaneously, IgE cross-linking resulted in downregulation of CD163, CD206 and MerTK (Figure 1), all regulatory scavenger receptors for haptoglobin–haemoglobin complexes, glycoproteins and apoptotic cells, respectively [41,42,43] (Figure 1). These receptors maintain a homeostatic, anti-inflammatory phenotype in monocytes, and are associated with tumour promoting functions such as invasion and immunosuppression [41,42,43]. When these markers are downregulated via proteolytic cleavage, this signifies pro-inflammatory activation of monocytes and potentially anti-tumour functions [41,42,43]. Together, these findings point to an IgE-induced monocyte activation, antigen presentation and pro-inflammatory profile.

This activatory surface marker signature induced by IgE cross-linking was accompanied by pro-inflammatory cytokine and chemokine secretion. TNFα, MCP-1 and IL-10, which we previously reported to be enhanced in the tumour microenvironment following systemic IgE treatment in vivo [12], were secreted by monocytes alongside immune mediators CXCL-10, IL-4, IL-1β and IL-23 (Figure 2). These mediators span across Th1/M1, Th17—anti-parasitic and infection clearance-related immune responses [44]—suggesting that in addition to mediating tumour cell killing, IgE cross-linking possibly enhances monocyte-mediated anti-tumour immunological cascades that employ different arms of immunity.

We also found that saturation of FcεRs by IgE did not induce higher levels of cytokine and chemokine release or enhanced FcεRI pathway signalling in comparison to cross-linking FcεRs partly-occupied by endogenous IgE. This observation is consistent with our previous findings showing maximal activation of human macrophages by IgE stimulation even in the absence of FcεR saturation [22]. Cross-linking of a proportion of IgE-FcεRI complexes on the cell surface has been shown to be sufficient for maximum mast cell and basophil activation and mediator release [45,46,47]. Monocytes already have FcεRs partly occupied by endogenous IgE and thus exogenous provision of MOv18 IgE will result in a fraction of FcεRs bearing tumour-specific IgE. However, this appears to be sufficient for triggering significant activation in monocytes, tumour cell killing by ADCC and cytokine release. Consistently, in our experiments, IgE-mediated stimulation of cytokine release by monocytes did not require the saturation of FcεRs. This property, likely unique to the IgE class, may be attributed to the very high-affinity and very slow dissociation rate of IgE-FcεR complexes [20]. Furthermore, it has been shown that there were no significant differences between the number of FcεRI receptors on monocytes in healthy volunteers and allergic patients [15]. Interestingly, recent reports suggest that patients with IgE deficiency have higher risk of developing malignancies [48,49]. It is possible that IgE deficient patients with cancer, whose IgE receptors may be mostly unoccupied, may also benefit from treatment with tumour antigen-specific IgE immunotherapy to prompt immune cell activation and cytotoxic killing of target tumour cells. Further investigation to assess endogenous IgE levels in cancer patients may lead to patient stratification for treatment.

We investigated the downstream signalling effects of monocyte activation by IgE cross-linking of the trimeric FcεRI. FcεR signalling in monocytes is not well-studied while IgE-mediated activation of the tetrameric FcεRI αβγ2 on the mast cell and basophil surface has been extensively researched. Monocytes express low levels of the trimeric FcεRI, αγ2. This isoform lacks the β-chain, reported to be responsible for enhanced downstream signalling and activation in mast cells [11,19,50]. Although it is reported that Lyn, a main orchestrator driving the FcεRI intracellular signal transduction cascade, binds to the FcεRI β-chain, here, we found that both Lyn and also Fyn, another FcεRI signalling protein, were phosphorylated upon IgE cross-linking on monocytes (Figure 3) [22,51,52,53,54,55]. STAT5, known to associate with Fyn and previously reported in mast cells and B cells, was also upregulated (Figure 3C) [56,57,58]. IgE stimulation also upregulated Yes and Lck, which although they are not members of the Src kinase family to which Lyn, Fyn and Fgr belong, they are described to sometimes substitute Fyn [28,29,30]. Together, this enabled us to augment the current understanding of monocyte-associated trimeric FcεRI signalling cascade with the addition of more recently-identified kinases such as Fgr, STAT5, Lck, Yes, and β-catenin (Figure 3, Table S1) [59]. Moreover, this depicts for the first time, Fgr, Lyn and Fyn associated with the functions of the γ2 FcεRI subunit [57,60,61]. Thus, despite lacking the high-signalling β-subunit, trimeric FcεRI on monocytes shares signalling cascades similar to those of the highly-expressed tetrameric FcεRI on mast cells and basophils. These functions may be orchestrated via the γ-chain of trimeric FcεRI, at least in monocytes [53,54,55]. Consistent with previously-reported transcriptomic analyses of tumours following treatment with IgE in vivo [23], we found heightened classical immune activation signalling pathways (e.g., FcγR signalling and functions, and CD28 co-stimulation signalling) following IgE cross-linking (Figure 3D). These results support the premise that monocytes are key IgE immune effector cells able to receive strong stimulating signals with IgE engagement.

Despite a slight decrease in FcεRI-expressing monocyte populations in patients compared to healthy subjects (Figure 4A), MOv18 IgE could direct both healthy volunteer- and ovarian cancer patient-derived monocytes to perform cytotoxic tumour cell killing (Figure 4B) [8,12]. In these co-cultures of tumour cells and monocytes, we detected upregulation of cytokines (TNFα, MCP-1, IL-10, CXCL-10, IL-1β, IL-6, IL-23) associated with MOv18 IgE ADCC compared to isotype control-triggered cytotoxicity. These mediators were similar to those produced with IgE cross-linking on monocytes (Figure 2A and Figure 4(Cii)). These signify the potential for IgE to configure an immunoactivatory milieu and to stimulate monocytes from cancer patients to secrete pro-inflammatory mediators alongside potentiating tumour cell death. Consistent with these findings, in a cohort of 1656 ovarian cancer patients [62], higher intratumoural expression of combined mediators found upregulated with monocyte activation by IgE (TNFα, MCP-1, IL-10, CXCL-10, IL-1β, IL-6 and IL-23) and in combination with key protein kinases downstream of FcεRI (LYN, FYN and FGR) and FcεRs were associated with more favourable five-year overall survival [12,44]. These results point to IgE-potentiated immune-activating and anti-tumour cascades in monocytes, potentially offering beneficial clinical effects.

In ex vivo functional studies, we aimed to understand the conditions required for the production of TNFα, MCP-1 and IL-10, consistently detected in IgE stimulated and ADCC supernatants. Cross-linking of IgE antibodies of different antigen specificity on monocytic cell surface triggered upregulation of TNFα (Figure 5A), consistent with previous reports by us and others [12,63]. Stimulating human monocytic cells and cancer cells with TNFα triggered increased MCP-1 (Figure 1C), as previously-reported with TNFα-stimulated human vascular endothelial cells [64,65]. TNFα-potentiated upregulation of the monocyte chemoattractant MCP-1 has been reported in IgE-mediated parasite response, and in the tumour microenvironment following systemic IgE treatment in vivo associated with significant recruitment of macrophages towards tumour lesions [8,12,15,23]. Furthermore, IL-10 was upregulated when human monocytic cells were either stimulated with combined TNFα and MCP-1 (Figure 5E), or in an autocrine manner by IL-10 (Figure 5F). Combined TNFα and MCP-1 stimulation of IL-10 production has not been previously reported, while IL-10-mediated autocrine stimulation was demonstrated in monocyte-derived macrophages [66]. IL-10 production was reported following IgE-initiated immune effector cell clearance of parasites [25]. It is possible that, following IgE-mediated activation of monocytes and macrophages, production of IL-10 may serve as a checkpoint to moderate the extent of heightened immune responses and limit local tissue damage.

Our study therefore provides evidence that IgE stimulates human monocytes towards a pro-inflammatory state and triggers immune mediator cascades and classical immune signalling pathways. These attributes can be directed against cancer cells and may contribute to the promise of IgE as a new candidate class for cancer immunotherapy.

## 4. Materials and Methods

### 4.1. Human Samples and Ethics

Venous blood samples were collected from healthy subjects and patients with ovarian cancer in K2EDTA tubes with informed written consent, in accordance with the Helsinki Declaration. The study design was approved by the Guy’s Research Ethics Committee (reference 09/H0804/45), Guy’s and St. Thomas’ NHS Foundation Trust. Peripheral blood samples were also purchased from the UK National Health Service (NHS) Blood and Transplant (BT) system from anonymous donor leukocyte cones. Monocyte isolation and flow cytometric analyses are described in Supplementary Materials.

### 4.2. Primary Monocyte Stimulation by IgE Cross-Linking

Primary monocytes were incubated at 1 × 10^6^ cells/mL with 5 µg/mL IgE, or media control, for 45 min at 37 °C. Following washing, cross-linking was stimulated with 5 µg/mL polyclonal goat anti-human IgE at 37 °C. For analysis of protein kinase phosphorylation profiles, cross-linking for 5 min was followed by immediate wash and cell lysis. For flow cytometric surface cell marker expression and soluble mediator analyses, cells were washed after 45 min cross-linking, re-suspended in fresh media (supplemented with 20 ng/mL M-CSF for surface cell marker expression), and incubated for 24 h at 37 °C. A Magnetic Luminex Performance Assay (Bio-Techne; LXSAHM) was performed to detect chemokine and cytokine release in cell culture supernatants, using a Luminex^®^ FlexMap 3D^®^ analyser. Serum IgE levels were analysed by Viapath Analytics (UK). Analysis of TNFα, MCP-1 and IL-10 mRNA expression (qPCR) and secretion (ELISA) assays using cell lines are described in Supplementary Materials.

### 4.3. Analysis of Phosphorylation Profile of Protein Kinases

The Proteome Profiler Human Phospho-Kinase Array (R&D Systems; ARY003B) was performed according to manufacturer’s instructions. Densitometric quantification was conducted using ImageJ software (National Institutes of Health, Bethesda, MD, USA).

### 4.4. Study of FcεRI Pathway and Implicated Pathways Based on Protein Kinase Analysis

Protein kinases that displayed significantly modulated phosphorylation (*p* < 0.05) upon IgE cross-linking compared to the baseline condition (untreated (endogenous IgE) vs. anti-IgE (endogenous IgE + anti-IgE); MOv18 IgE vs. MOv18 IgE + anti-IgE) were mapped based on previously reported data, new findings in this study, and the FcεRI signalling pathway as described in the Kyoto Encyclopedia of Genes and Genomes (KEGG) database [59]. When inferring implicated pathways, genes of kinases with at least 20% change in phosphorylation upon IgE cross-linking were considered. Pathway enrichment was calculated through the enrichPathway v.1.30.0 function [67] in R v.3.6.1, which incorporates a hypergeometric model [68] to assess whether the number of selected kinase genes are enriched in Reactome pathways. Pathways were selected with *p*-value cut-off of 0.01 and q-value 0.2. To derive immunologically-relevant pathways, enriched pathways were curated to include a minimum gene set size of 2, maximum gene set of 134 genes and a ratio of differentially phosphorylated genes to total genes in the pathway equal to 0.029 or higher.

### 4.5. Statistical Methods and Survival Analyses

All statistical analyses were performed using GraphPadTM Prism Software (version 8.0). Error bars represent standard error of mean (SEM). Clinical associations of intratumoural gene expression of mediators were assessed using publicly-available data in Kaplan–Meier plotter (http:/kmplot.com/analysis/) as described previously [44], between two patient cohorts: patients with top 25% and bottom 25% expression of mediators within the tumours. Student’s *t*-test or a one-way ANOVA with Tukey’s post-test were used to determine statistical significance of the data.

## 5. Conclusions

IgE can activate human monocytes to acquire a pro-inflammatory phenotype capable of mediating effector functions against tumour cells through monocyte-specific FcεRI intracellular pathway signalling. These insights deepen our understanding on the mechanism of IgE-based cancer immunotherapy and may further contribute to the development of novel treatments for solid tumours.

## Supplementary Materials

The following are available online at https://www.mdpi.com/2072-6694/12/11/3376/s1, Figure S1: FcεR expression on monocytes detected by flow cytometry, Figure S2: Immune mediators secreted upon IgE cross-linking on monocytes, Table S1: Kinases detected downstream of FcεRI-signalling upon IgE cross-linking on monocytes in the KEGG database, Table S2: List of genes, number of selected genes, and total number of genes in implicated pathways upon IgE cross-linking, detected through Reactome pathway enrichment, Table S3: Clinical characteristics of healthy volunteers (*n* = 34) and ovarian cancer patients (*n* = 110) used for evaluation of total serum IgE, Figure S3: Immune mediators secreted upon IgE-mediated tumour cell killing with monocytes, Figure S4: IL-10 stimulates IL-10 expression in a positive feedback loop.
