# Supplementary material for: IgE Activates Monocytes from Cancer Patients to Acquire a Pro-Inflammatory Phenotype
Source: Cancers (Basel). 2020 Nov 15;12(11):3376. doi: 10.3390/cancers12113376 (PMC7698027; doi:10.3390/cancers12113376)
Supplement: Supplementary file 1 [file cancers-12-03376-s001.pdf]

Supplementary Materials:

IgE Activates Monocytes from Cancer Patients to Acquire a Pro-Inflammatory Phenotype

Mano Nakamura, Elmira Amiri Souri, Gabriel Osborn, Roman Laddach, Jitesh Chauhan, Chara Stavraka, Sara Lombardi, Anna Black, Atousa Khiabany, Duaa O. Khair, Mariangela Figini, Anna Winship, Sharmistha Ghosh, Ana Montes, James F. Spicer, Heather J. Bax, Debra H. Josephs, Katie E. Lacy, Sophia Tsoka and Sophia N. Karagiannis

Isolation of Primary Monocytes from Blood Samples

Peripheral blood monocytes were isolated using the RosetteSep™ Human Monocyte Enrichment Cocktail (STEMCELL™ Technologies, 15068). Monocytes from leukocyte cones were purified by first using the Ficoll density gradient centrifugation method to isolate PBMCs, then the magnetic column separation method to purify monocytes (Pan-Monocyte Isolation Kit, LS column, and a MACS Multistand (MACS Miltenyi Biotec; 130-096-537, 130-042-401, 130-043-303) according to the manufacturer's instructions. The analysis of phosphorylation profile of protein kinases and cytokine/chemokine secretion upon IgE cross-linking utilized monocytes isolated from leukocyte cones.

Cell Lines

Human monocytic U937 cells (CRL-1593.2), IGROV1 human ovarian carcinoma cells expressing human FRα [36,62] and A375 human metastatic melanoma cells (CRL-11147, ATCC) were grown using appropriate medium, supplemented with 10% FCS. Routine mycoplasma testing was performed by PCR. Cells were maintained in a Nuaire™ CO_2 air-jacketed incubator, at 37 °C in 5% CO_2.

IgE Cross-Linking and Cytokine Stimulation by Monocytic and Tumor Cells

Prior to IgE cross-linking or cytokine stimulation, U937 monocytes were primed for 48 h with 50 ng/mL IL-4, to upregulate CD23 cell surface expression, then were passaged and re-stimulated with IL-4 for a further 48 h. U937, IGROV1, and A375 cells were plated at a density of 1×10^6 cells/mL in a 24-well plate (0.5×10^6 cells per well).

For IgE cross-linking, U937 cells were stimulated with 5µg/mL IgE, or media control, for 1 hour at 37 °C. Following washing, cross-linking was stimulated with 5µg/mL polyclonal goat anti-human IgE at 37 °C for 1 h. Cells were washed and resuspended in RLT buffer for RNA isolation by RNeasy Kit (Qiagen; 74106).

For cytokine stimulation, TNFα, MCP-1, or IL-10 were added separately in each well at final concentrations of 10 ng/mL or combinations of TNFα and MCP-1 at final concentrations of 10 ng/mL each. Cells were incubated at 37 °C for 3 h for gene expression analysis by qPCR, or for 10 h for cytokine secretion analysis by ELISA. For qPCR analysis, cells were washed and resuspended in RLT buffer for RNA isolation by RNeasy Kit (Qiagen; 74106).

Flow Cytometric Analyses of Cell Surface Markers

To identify monocytes, Fc receptor Block (BioLegend; 422302), LIVE/DEAD stain (Invitrogen; L10119), anti-CD14-PE (clone: M5E2; BioLegend; 301806) and anti-CD16-BV510 (clone: 3GB; BioLegend; 302048) were used. To gate out the non-monocytes, anti-CD3- PerCP/Cy5.5 (clone: UCHT1; BioLegend; 300430), anti-CD19-BV421(PB) (clone: HIB19; BioLegend; 302234), and anti-CD56-PE/Cy7 (clone: HCD56; BioLegend; 318318) were used to exclude the T, B, and NK cells, respectively. For surface protein expression: anti-CD40-BV510 (clone: 5C3; BioLegend; 334330), anti-CD80-BUV395

(clone: L307.4; BD; 565210), anti-CD86-BUV737 (clone: 2331 (FUN-1); BD; 612784), anti-CD163-APC/Fire™ 750 (clone: GHI/61; BioLegend; 333634), anti-CD206-FITC (clone: 15-2; BioLegend; 321104), and anti-MerTK-BV711 (clone: 590H11G1E3; BioLegend; 357620) were used. 1×10^5 cells were incubated with 1µL of each antibody for 20 min at 4 °C, washed and 2×10^4 viable monocytes were acquired and analysed using a BD FACS Canto™ II and BD LSRFortessa™. Flow cytometric dot plots were analysed on FlowJo (TreeStar Inc.) software.

Tumor Cell Cytotoxicity and Phagocytosis Assay

Antibody-dependent cellular cytotoxicity and phagocytosis (ADCC/ADCP) of IGROV1 cells was quantified by adapting a previously-described three-colour flow cytometric method [24]. Primary monocytes isolated from healthy volunteers and cancer patients (effector cells) were incubated with IGROV1 (target cells), and 5µg/mL antibodies (Effector:Target cell ratio 3:1).

Analysis of TNFα, MCP-1, and IL-10 Expression

qPCR was used to analyze the TNFα, MCP-1, and IL-10 mRNA expression following stimulation with cytokines or IgE. RNA was isolated by RNeasy Kit, cDNA was synthesized (Thermo Fisher Scientific; 4368814), and PCR was completed using TaqMan Gene Expression Assay probes: TNFα FAM (Hs01113624_g1)(Thermo Fisher Scientific; 4351370), MCP-1 FAM (Hs00234140_m1) (Thermo Fisher Scientific; 4351370), IL-10 FAM (Hs00961622_m1)(Thermo Fisher Scientific; 4351368), and GAPDH VIC (Hs99999905_m1)(Thermo Fisher Scientific; 4448485) for house-keeping gene control. qPCR was run in technical triplicates. Fold-change in target mRNA expression was calculated in relation to the unstimulated condition.

Cytokine ELISA

TNFα, MCP-1 and IL-10 in cell culture supernatants were measured using cytokine sandwich enzyme-linked immunosorbent assays (R&D Systems; DY210-05, DY279B-05, DY217B-05) following the manufacturer's instructions. Plates were read using a Flurostar® Omega Spectrophotometer (BMG Labtech).

Supplementary Figures and Tables

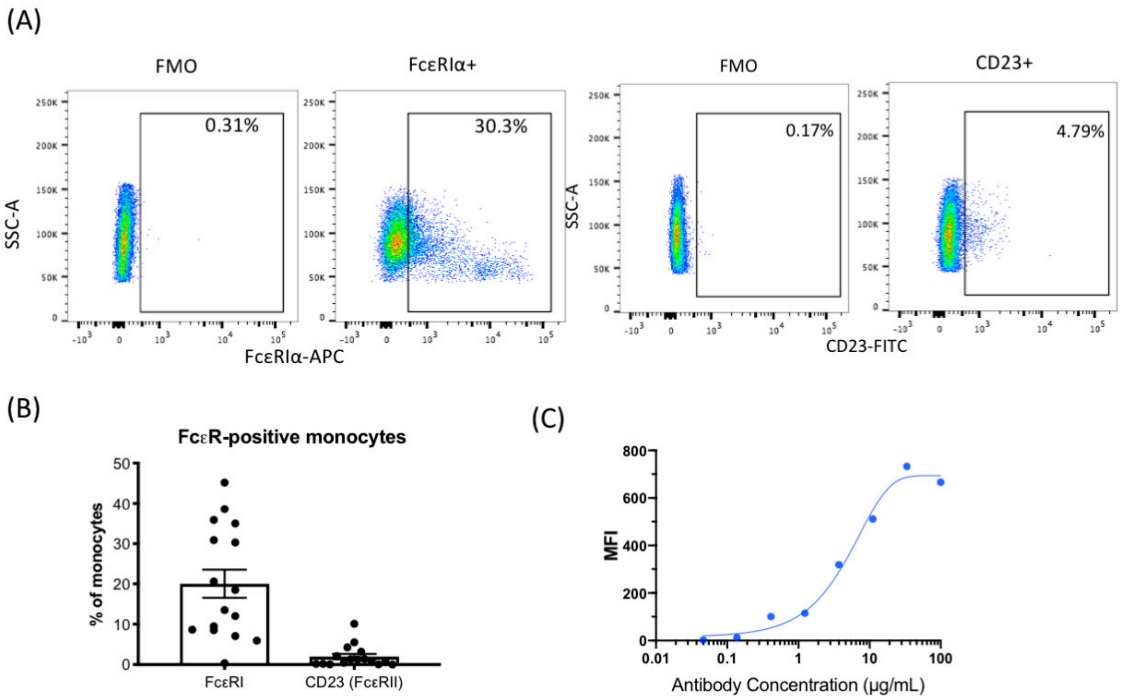

Figure S1. FcεR expression on monocytes detected by flow cytometry. (**A**) Representative flow cytometry dot-plots of FcεRI – (left) and CD23- positive (right) monocytes (n=16). (**B**) Average proportion of FcεR-expressing (FcεRI, CD23) healthy volunteer monocytes (n = 16). Error bars represent standard error of mean (SEM). (**C**) Change in monocyte FcεR occupancy following stimulation with MOv18 IgE (n = 1).

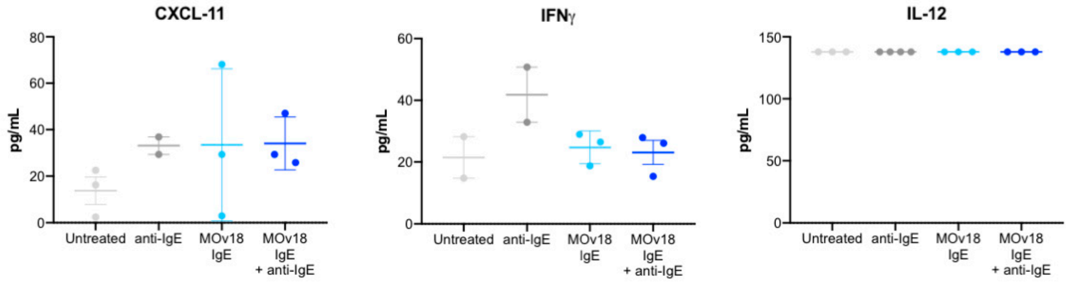

Figure S2. Immune mediators secreted upon IgE cross-linking on monocytes. Additional cytokines and chemokines (Luminex) measured in cell culture supernatants following cross-linking of IgE on the surface of primary monocytes isolated from healthy volunteer blood. Error bars represent standard error of mean (SEM) of n = 3 independent experiments. A student's t-test was performed to assess significance.

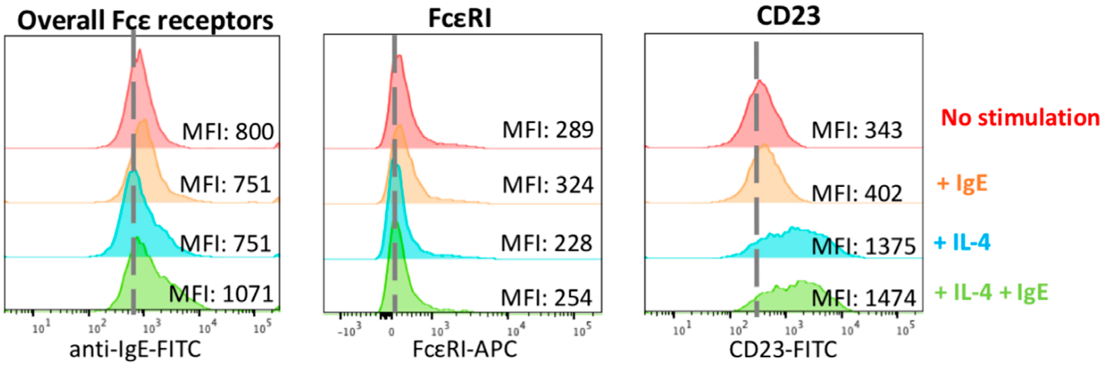

Figure S3. Exposure to IgE does not alter FcεRI levels on human monocytes. FcεR expression of primary monocytes isolated from healthy volunteers following 24 h stimulation with no stimulation, IgE, IL-4 or a combination of IgE and IL-4 stimulation (n = 1).

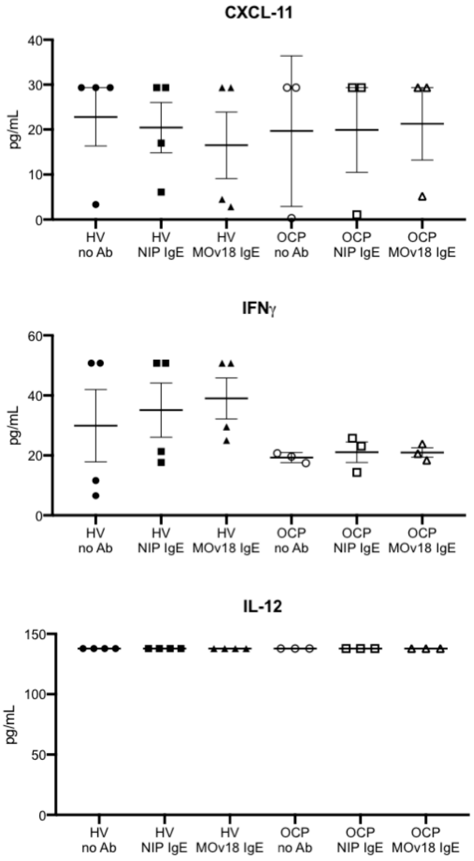

Figure S4. Immune mediators secreted upon IgE-mediated killing of tumour cells by human monocytes. Additional cytokine and chemokines (Luminex) measured in cell culture supernatants from IgE-mediated ADCC/ADCP assays with primary monocytes isolated from healthy volunteers (HV) (n = 4) and from ovarian cancer patients (OCP) (n = 3) (independent experiments). Error bars represent standard error of mean (SEM). A One way-ANOVA with Tukey's post-test was performed to assess significance.

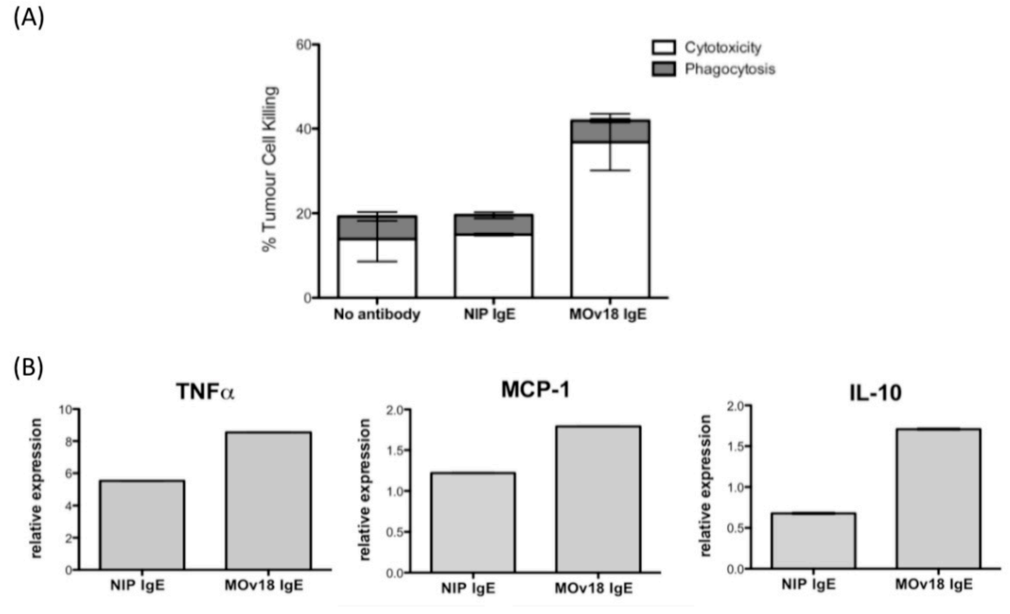

Figure S5. TNFα, MCP-1 and IL-10 expression was upregulated by U937 monocytic cells following MOv18 IgE-dependent cytotoxic killing of target IGROV1 tumour cells. (**A**) MOv18 IgE potentiated in vitro killing of target IGROV1 ovarian cancer cells (compared with no antibody (no Ab) and isotype (NIP IgE) controls) by U937 monocytic cells (n = 2). Error bars represent standard deviation (SD). (**B**) Relative mRNA expression of TNFα, MCP-1 and IL-10 by human monocytic U937 cells following IgE-dependent cytotoxic killing of target IGROV1 cancer cells (n = 1).

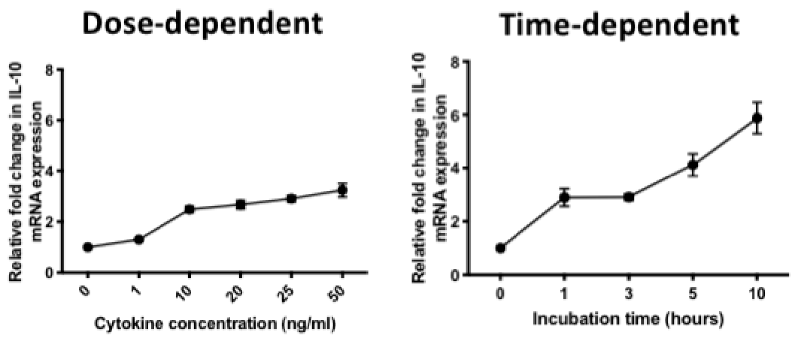

Figure S6. IL-10 stimulates IL-10 expression in a dose- and time-dependent manner. IL-10 mRNA expression by U937 monocytic cells following IL-10 stimulation in increasing doses (0–20ng/mL, n = 5) and incubation times (0–10 h, n = 5) (independent experiments). Error bars represent standard error of mean (SEM).

Table S1. Kinases detected downstream of FcεRI-signaling upon IgE cross-linking on monocytes, in the KEGG database.

			**Endogenous IgE Cross-Linking (Untreated** vs **Anti-IgE)**		**Cross-Linking Following Saturation with Exogenous IgE (MOv18 IgE vs MOv18 IgE + Anti-IgE)**	
Pathway Name	Total number of genes in pathway (path)	Number of genes in the pathway analyzed	Name of genes that appear perturbed in the pathway (gene) (number of selected genes)	Gene_path_ratio (gene/path)	Name of genes that appear perturbed in the pathway (gene) (number of selected genes)	Gene_path_ratio (gene/path)
FcεRI signaling currently on KEGG	68	12	p38a/ERK1/ERK2/ AKT1/AKT2/AkT3/ Lyn/Fyn (8)	0.12	ERK1/ERK2/AKT1/ AKT2/AkT3/ Lyn/Fyn (7)	0.10
FcεRI signaling currently on KEGG + newly associated kinases	73	17	p38a/ERK1/ERK2/AKT1/AKT2/AKT3/ Lyn/Fyn/ Fgr[†55,56/] STAT5a[†53,54]/STAT5b[†53,54] (11)	0.15	ERK1/ERK2/AKT1/ AKT2/AKT3/ Lyn/Fyn/ Fgr[†55,56]/ STAT5a[†53,54]/STAT5b[†53,54]/ Yes[†33,34]/Lck[†32,33]/ (12)	0.16

†Newly associated kinases downstream of FcεRI signaling, based on data shown in Figure 3 and recent literature as referenced within the table.

Table S2. List of genes, number of selected genes, and total number of genes in implicated pathways upon IgE cross-linking, detected through Reactome pathway enrichment.

			**Endogenous IgE Cross-Linking (Untreated vs Anti-IgE)**		**Cross-Linking Following Saturation with Exogenous IgE (MOv18 IgE vs MOv18 IgE + Anti-IgE)**	
	Pathway Name	Total number of genes in pathway (path)	Number of genes in the pathway analyzed	Name of genes that appear perturbed in the pathway (gene) (number of selected genes)	Gene_path_ratio (gene/path)	Name of genes that appear perturbed in the pathway (gene) (number of selected genes)
1	FcγR activation	12	Src/Lyn/Fyn/Fgr/Hck (5)	0.42	Lyn/Fyn/Yes/Fgr (4)	0.33
2	CTLA-4 inhibitory signaling	21	Akt 1/Akt 2/Akt 3/Src/Lyn/Fyn (6)	0.29	Akt 1/Akt 2/Akt 3/Lyn/Lck/Fyn/Yes (7)	0.33
3	CD28 co-stimulation	33	Akt 1/Akt 2/Akt 3/TOR/Src/Lyn/Fyn (7)	0.21	Akt 1/Akt 2/Akt 3/Lyn/Lck/Fyn/Yes (7)	0.21
4	IL-21 signaling	10	STAT5a/STAT5b (2)	0.20	STAT5a/STAT5b/STAT3 (3)	0.30
5	IL-2 signaling	12	STAT5a/STAT5b (2)	0.17	Lck/STAT5a/STAT5b/PYK2 (4)	0.33
6	IL-15 signaling	14	STAT5a/STAT5b (2)	0.14	STAT5a/STAT5b/STAT3 (3)	0.21
7	IL-3, IL-5 and GM-CSF signaling	48	Lyn/STAT5a/Fyn/STAT5b/Hck (5)	0.10	Lyn/STAT5a/Fyn/Yes/STAT5b (5)	0.10
8	CD209 (DC-SIGN) signaling	21	Lyn/Fyn (2)	0.10	MSK1/Lyn/Fyn (3)	0.14
9	FcεRI mediated MAPK activation	32	ERK1/ERK2/Lyn (3)	0.09	ERK1/ERK2/Lyn (3)	0.09
10	Negative regulation of the PI3K/AKT network	110	ERK1/ERK2/EGFR/Akt 1/Akt 2/Akt 3/Src/Fyn/PDGFR-β (9)	0.08	ERK1/ERK2/EGFR/Akt 1/Akt 2/Akt 3/Lck/Fyn (8)	0.07
11	FcγR-dependent phagocytosis	86	ERK1/ERK2/Src/Lyn/Fyn/Fgr/Hck (7)	0.08	ERK1/ERK2/Lyn/Fyn/Yes/Fgr (6)	0.07
12	IL-20 family signaling	25	STAT5a/STAT5b (2)	0.08	STAT5a/STAT5b/STAT3 (3)	0.12
13	VEGF signaling	107	p38a/Akt 1/Akt 2/Akt 3/TOR/β-catenin/Src/Fyn (8)	0.07	Akt 1/Akt 2/Akt 3/β-catenin/Fyn/eNOS/PYK2 (7)	0.07
14	FcεRI signaling	134	ERK1/ERK2/Lyn/Fyn (4)	0.03	ERK1/ERK2/Lyn/Fyn (4)	0.03

Table S3. Clinical characteristics of healthy volunteers (n = 34) and ovarian cancer patients (n = 110) used for evaluation of total serum IgE levels (Figure 4A (iv)).

	**Healthy Volunteers**	**Ovarian Cancer Patients**
n-number	n = 34	n = 110
Average age ± SEM	49.79 ± 2.92	62.65 ± 1.12
Female (%)	100%	100%
Stage 1	-	14.55%
Stage 2	-	7.27%
Stage 3	-	60.91%
Stage 4	-	15.45%
Unknown Stage		1.82%
